# Octyl (2*E*)-2-[2-(di­phenyl­phosphan­yl)benzyl­idene]hydrazinecarbodi­thio­ate

**DOI:** 10.1107/S1600536814008459

**Published:** 2014-05-03

**Authors:** Izuddin Asri, Malai Haniti S. A. Hamid, Aminul Huq Mirza, Mohammad Akbar Ali, Mohammad Rezaul Karim

**Affiliations:** a ChemicalStudies, Faculty of Science, University of Brunei Darussalam, Jalan Tungku Link, Gadong, BE 1410, Brunei; b Department of Chemistry, Boswell Science Complex, Tennessee State University, Nashville, 3500 John A Merritt Blvd, Nashville, TN 37209, USA

## Abstract

The title compound, C_28_H_33_N_2_S_2_P, adopts the thione tautomeric form, as supported by the C—S distance [1.6744 (18) Å]. The Schiff base exhibits an *E* conformation about the C=N bond but a *Z* conformation about the C—N bond. The terminal chain is disordered over two sets of sites with an occupancy ratio of 0.732 (3):0.268 (3). In the crystal, pairs of N—N—H hydrogen bonds between the thione groups link neighbouring mol­ecules into centrosymmetric dimers.

## Related literature   

For Schiff bases derived from S-alk­yl/aryl esters of di­thio­carbazic acid, see: Akbar Ali *et al.* (2012 ▶, 2013 ▶); Hamid *et al.* (2009 ▶); Akbar Ali *et al.* (2005 ▶). For their chemotherapeutic properties, see: Tarafder *et al.* (2002 ▶); Akbar Ali & Livingstone (1974 ▶); Akbar Ali *et al.* (2002 ▶); Hossain *et al.* (1996 ▶). For related structures, see: Su *et al.* (1999 ▶); Song *et al.* (2009 ▶); Shanmuga Sundara Raj *et al.* (2000 ▶). For standard bond lengths, see: Allen *et al.* (1987 ▶).
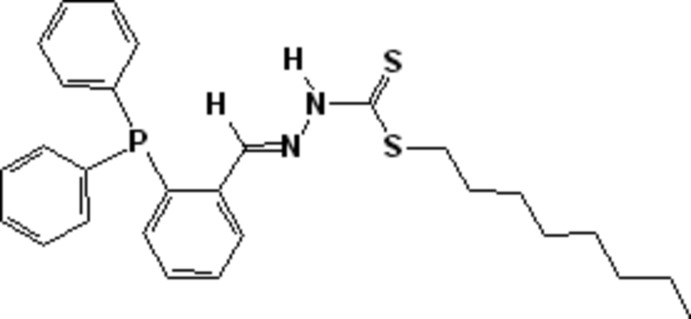


## Experimental   

### 

#### Crystal data  

C_28_H_33_N_2_PS_2_
*M* *_r_* = 492.65
Triclinic, 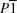

*a* = 11.2068 (12) Å
*b* = 11.4956 (12) Å
*c* = 11.7728 (13) Å
α = 86.623 (1)°
β = 70.538 (1)°
γ = 65.013 (1)°
*V* = 1290.2 (2) Å^3^
*Z* = 2
Mo *K*α radiation
μ = 0.29 mm^−1^
*T* = 100 K
0.60 × 0.60 × 0.38 mm

#### Data collection  

Bruker APEXII CCD diffractometer
Absorption correction: multi-scan (*SADABS*; Sheldrick, 2002 ▶) *T*_min_ = 0.846, *T*_max_ = 0.899
15526 measured reflections
5343 independent reflections
4954 reflections with *I* > 2σ(*I*)
*R*_int_ = 0.025

#### Refinement  

*R*[*F* ^2^ > 2σ(*F* ^2^)] = 0.043
*wR*(*F* ^2^) = 0.111
*S* = 1.04
5343 reflections
356 parameters
209 restraints
H atoms treated by a mixture of independent and constrained refinement
Δρ_max_ = 1.30 e Å^−3^
Δρ_min_ = −0.51 e Å^−3^

### 

Data collection: *APEX2* (Bruker, 2007 ▶); cell refinement: *SAINT* (Bruker, 2007 ▶); data reduction: *SAINT*; program(s) used to solve structure: *SHELXS97* (Sheldrick, 2008 ▶); program(s) used to refine structure: *SHELXL97* (Sheldrick, 2008 ▶); molecular graphics: *SHELXTL* (Sheldrick, 2008 ▶); software used to prepare material for publication: *SHELXTL* and *publCIF* (Westrip, 2010 ▶).

## Supplementary Material

Crystal structure: contains datablock(s) I, New_Global_Publ_Block. DOI: 10.1107/S1600536814008459/bq2394sup1.cif

Structure factors: contains datablock(s) I. DOI: 10.1107/S1600536814008459/bq2394Isup2.hkl

Supporting information file. DOI: 10.1107/S1600536814008459/bq2394Isup3.cml

CCDC reference: 997277

Additional supporting information:  crystallographic information; 3D view; checkCIF report

## Figures and Tables

**Table 1:** Hydrogen-bond geometry (Å, °)

*D*—H⋯*A*	*D*—H	H⋯*A*	*D*⋯*A*	*D*—H⋯*A*
N2—H2*N*⋯S2^i^	0.90 (2)	2.45 (2)	3.3337 (17)	168 (2)

Symmetry code: (i) 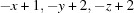.

**Fig. 1.**
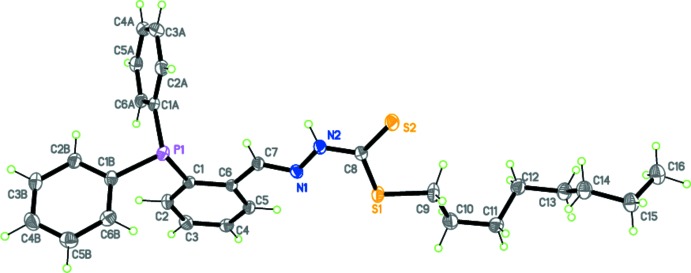
ORTEP diagram of the title compound only with the major part of the disordered terminal chain.

**Fig. 2.**
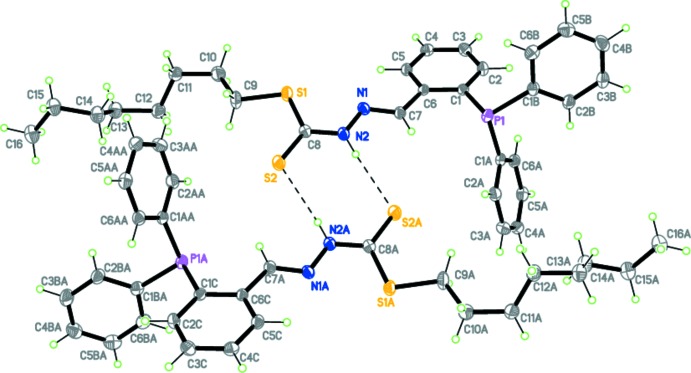
H-bonded centrosymmetric dimers of the title compound.

## supplementary crystallographic information

### 1. Comment

In recent years, considerable attention has been focused on Schiff bases derived from S-alkyl/aryl esters of dithiocarbazic acid (M. Akbar Ali *et al.* 2013, M. Akbar Ali *et al.* 2012) as they belong to a promising class of potentially bioactive chelating agents containing mixed hard and soft donor atoms. These organic chelators could also lead to the formation of coordination compounds with useful chemotherapeutic properties (Tarafder *et al.*, 2002; Akbar Ali *et al.*, 2002; Akbar Ali *et al.*, 1974; Hossain *et al.*, 1996). In view of less crystallographic data available on Schiff bases containing mixed hard and soft donor atoms such as nitrogen, sulfur and phosphorus, the new Schiff base, C_28_H_33_N_2_S_2_P (I) was synthesized by the reaction of S-octyl dithiocarbazate with 2-(diphenylphosphino)benzaldehyde in ethanol. The crystal is triclinic, space group P-1. The asymmetric unit contains one molecule of the compound C_28_H_33_N_2_S_2_P. The terminal chain C11 to C16 is disordered into two positions with occupancy ratio = 73:27. Restraints in bond lengths and thermal parameters were applied to the disordered parts. H atom of N2 was located from different map and refined with restraints in bond length and thermal parameters. Final R values are R1 = 0.0429 and wR2 = 0.1084 for 2-theta up to 55°. Like most Schiff bases derived from S-alkyl/aryl esters of dithiocarbazic acid, the Schiff base, I also remains in its thione tautomeric form as supported by the C8-S2 distance [1.6744 (18) Å], which is typical of double bonds and are by far the shortest C-S distances observed so far among the Schiff bases derived from S-alkyl/aryl dithiocarbazates (Hamid *et al.* 2009, Akbar Ali *et al.* 2005). The Schiff base also remains in an E configuration about the C7-N1 bond but a Z configuration about the C8-N2 bond. The C7-N1 bond distance compares well with that of the C=N double bonds in other related compounds (Su *et al.* 1999, Song *et al.* 2009). The C8-N2 bond distance [1.337 (2) Å] indicates that the N2 nitrogen atom is *sp^2^* hybridized and the bond is closer to a double than a single bond (Allen *et al.* 1987). The N1–N2 bond in the Schiff base is shorter than a single N-N bond. A comparison of the N1—N2 distance [1.378 (2) Å] with that in S-benzyl dithiocarbazate [1.406 (3) Å] (Shanmuga Sundara Raj *et al.* 2000) indicates that there is a significant *π*-charge delocalization along the C-N-N-C chain. There is intermolecular hydrogen bonding between the hydrazine nitrogen atom N(2) of one molecule and the thione sulfur atom S2 of another molecule ( Fig. 2) resulting in H-bonded centrosymmetric dimers. The H-bonding stabilizes the E and Z conformations about the C7-N1 and C8-N2 bonds, respectively.

### 2. Experimental

A hot solution of *S*-n-octyldithiocarbazate (0.16 g, 0.7 mmol) in absolute ethanol (10 ml) was mixed with a solution of 2-di(phenylphosphino) benzaldehyde (0.21 g, 0.71 mmol) in the same solvent (5 ml). The resulting mixture was heated on a steam bath for 15 minutes and then left to cool. The yellow crystals that had formed were filtered off, recrystallized from an ethanol/chloroform mixture and dried *in vacuo*. Yield = 0.14 g (90%); m.p. = 114–116 °C; IR (cm^-1^): 3106 (N—H), 3068, 3048 (=CH, Ar), 1560 (C=N), 1024 (C—S), 1097 (C=S); UV-Vis: λ_max_/nm, (log ε (dm^3^ cm^-1^ mol^-1^): 374(5.369); ^1^H NMR (p.p.m., CDCl_3_): 10.23 (1*H*, s, –NH), 8.64 (1*H*, d, CH=N), 8.13 (1*H*, d, Ar), 7.66 – 7.22 (12*H*, m, Ar), 6.91 (1*H*, m, Ar), 3.25 (2*H*, t, CH_2_), 1.77 – 1.22 (12*H*, m, aliphatic protons), 0.88 (3*H*, t, CH_3_); ^13^ C NMR [p.p.m., CDCl_3_]: 199.6 (C=S), 143.7 – 126.7 (C=N, Ar—C), 34.7 (–CH2—C=S), 31.8, 29.2, 29.2, 29.1, 28.5, 22.7 (6xCH_2_), 14.1 (–CH_3_); Anal.calcd. for C_28_H_33_N_2_S_2_P: C 68.26, H 6.75, N 5.69; Found (%) C 69.35, H 6.15 N 5.68; MS/EI, m/*z* (I,%) for C_28_H_33_N_2_S_2_P (481.59 g/mol): 304.1 [M±C=SSOctyl] (17), 288.1 [M±NHC=SSOctyl] (100), 183.0 [PPh2] (22), 146.1 [SOctyl] (5).

The IR spectrum was recorded as KBr disc on a Perkin-Elmer 1600 F T IR spectrometer. The 1H NMR spectrum was run in CDCl3 on a Bruker Advance, 400 MHz spectrometer in the Department of Chemistry, Tennessee State University, USA. Elemental analysis for C, H and N was done by the Elemental Analysis Laboratory, Department of Chemistry, National University of Singapore. The EI mass spectrum was recorded on an Agilent Mass Spectrometer 5975 C MSD (with direct probe). The X-ray data were collected at the X-ray Diffraction Laboratory, Department of Chemistry, National University of Singapore using a Bruker-AXS Smart Apex CCD single-crystal diffractometer.

### 3. Refinement

The terminal chain C11 to C16 was disordered into two positions with occupancy ratio = 73:27. Restraints in bond lengths and thermal parameters were applied to the disordered parts. H atom of N2 was located from different map and refined with restraints in bond length and thermal parameters. Other H atoms were placed at calculated positions and were treated as riding on the parent C atoms with C—H = 0.95–0.99 Å, Uiso(H) = 1.2Ueq(C). Final *R* values are R1 = 0.0429 and wR2 = 0.1089 for 2-theta up to 55°.

### Crystal data

**Table:**

C_28_H_33_N_2_PS_2_	*Z* = 2
*M**_r_* = 492.65	*F*(000) = 524
Triclinic, *P*1	*D*_x_ = 1.268 Mg m^−^^3^
Hall symbol: -P 1	Mo *K*α radiation, λ = 0.71073 Å
*a* = 11.2068 (12) Å	Cell parameters from 5343 reflections
*b* = 11.4956 (12) Å	θ = 2.2–28.3°
*c* = 11.7728 (13) Å	µ = 0.29 mm^−^^1^
α = 86.623 (1)°	*T* = 100 K
β = 70.538 (1)°	Block, yellow
γ = 65.013 (1)°	0.60 × 0.60 × 0.38 mm
*V* = 1290.2 (2) Å^3^	

### Data collection

**Table:**

Bruker APEXII CCD diffractometer	5343 independent reflections
Radiation source: fine-focus sealed tube	4954 reflections with *I* > 2σ(*I*)
Graphite monochromator	*R*_int_ = 0.025
φ and ω scans	θ_max_ = 26.5°, θ_min_ = 1.8°
Absorption correction: multi-scan (*SADABS*; Sheldrick, 2002)	*h* = −14→14
*T*_min_ = 0.846, *T*_max_ = 0.899	*k* = −14→14
15526 measured reflections	*l* = −14→14

### Refinement

**Table:**

Refinement on *F*^2^	Primary atom site location: structure-invariant direct methods
Least-squares matrix: full	Secondary atom site location: difference Fourier map
*R*[*F*^2^ > 2σ(*F*^2^)] = 0.043	Hydrogen site location: inferred from neighbouring sites
*wR*(*F*^2^) = 0.111	H atoms treated by a mixture of independent and constrained refinement
*S* = 1.04	*w* = 1/[σ^2^(*F*_o_^2^) + (0.0483*P*)^2^ + 1.3326*P*] where *P* = (*F*_o_^2^ + 2*F*_c_^2^)/3
5343 reflections	(Δ/σ)_max_ = 0.001
356 parameters	Δρ_max_ = 1.30 e Å^−^^3^
209 restraints	Δρ_min_ = −0.51 e Å^−^^3^

### Special details

**Table:**

Geometry. All e.s.d.'s (except the e.s.d. in the dihedral angle between two l.s. planes) are estimated using the full covariance matrix. The cell e.s.d.'s are taken into account individually in the estimation of e.s.d.'s in distances, angles and torsion angles; correlations between e.s.d.'s in cell parameters are only used when they are defined by crystal symmetry. An approximate (isotropic) treatment of cell e.s.d.'s is used for estimating e.s.d.'s involving l.s. planes.
Refinement. Refinement of *F*^2^ against ALL reflections. The weighted *R*-factor *wR* and goodness of fit *S* are based on *F*^2^, conventional *R*-factors *R* are based on *F*, with *F* set to zero for negative *F*^2^. The threshold expression of *F*^2^ > σ(*F*^2^) is used only for calculating *R*-factors(gt) *etc*. and is not relevant to the choice of reflections for refinement. *R*-factors based on *F*^2^ are statistically about twice as large as those based on *F*, and *R*-factors based on ALL data will be even larger.

### Fractional atomic coordinates and isotropic or equivalent isotropic displacement parameters (Å^2^)

**Table:**

	*x*	*y*	*z*	*U*_iso_*/*U*_eq_	Occ. (<1)
P1	−0.07934 (5)	1.15477 (4)	1.32348 (4)	0.01927 (12)	
S1	0.51929 (5)	0.64877 (4)	0.91914 (4)	0.02410 (13)	
S2	0.66997 (5)	0.81091 (5)	0.92999 (5)	0.02737 (13)	
N1	0.28051 (15)	0.86048 (15)	1.05097 (14)	0.0193 (3)	
N2	0.39950 (16)	0.87988 (15)	1.02654 (14)	0.0207 (3)	
C1	−0.09012 (18)	1.03671 (16)	1.23193 (15)	0.0172 (3)	
C2	−0.21642 (19)	1.02936 (18)	1.24981 (17)	0.0219 (4)	
H2	−0.2996	1.0883	1.3089	0.026*	
C3	−0.2230 (2)	0.93807 (19)	1.18325 (17)	0.0230 (4)	
H3	−0.3103	0.9358	1.1961	0.028*	
C4	−0.1020 (2)	0.84996 (18)	1.09786 (16)	0.0214 (4)	
H4	−0.1060	0.7867	1.0526	0.026*	
C5	0.02430 (19)	0.85474 (17)	1.07898 (16)	0.0199 (4)	
H5	0.1070	0.7942	1.0206	0.024*	
C6	0.03213 (18)	0.94745 (16)	1.14455 (15)	0.0165 (3)	
C7	0.16737 (18)	0.95264 (17)	1.11708 (16)	0.0187 (4)	
H7	0.1710	1.0248	1.1485	0.022*	
C8	0.52454 (19)	0.78826 (17)	0.96203 (16)	0.0199 (4)	
C1A	−0.13142 (18)	1.30012 (17)	1.24425 (15)	0.0187 (4)	
C2A	−0.0523 (2)	1.37020 (19)	1.22768 (17)	0.0242 (4)	
H2A	0.0230	1.3421	1.2581	0.029*	
C3A	−0.0827 (2)	1.4809 (2)	1.16693 (19)	0.0289 (4)	
H3A	−0.0281	1.5279	1.1561	0.035*	
C4A	−0.1920 (2)	1.52279 (19)	1.12225 (18)	0.0271 (4)	
H4A	−0.2129	1.5985	1.0810	0.033*	
C5A	−0.2713 (2)	1.45323 (19)	1.13810 (17)	0.0249 (4)	
H5A	−0.3465	1.4817	1.1075	0.030*	
C6A	−0.24120 (19)	1.34284 (18)	1.19809 (16)	0.0218 (4)	
H6A	−0.2956	1.2957	1.2080	0.026*	
C1B	−0.23181 (19)	1.18714 (18)	1.46050 (16)	0.0208 (4)	
C2B	−0.3553 (2)	1.30064 (19)	1.49289 (18)	0.0265 (4)	
H2B	−0.3669	1.3667	1.4396	0.032*	
C3B	−0.4615 (2)	1.3177 (2)	1.60282 (19)	0.0316 (5)	
H3B	−0.5449	1.3957	1.6243	0.038*	
C4B	−0.4468 (2)	1.2223 (2)	1.68103 (18)	0.0303 (4)	
H4B	−0.5199	1.2341	1.7557	0.036*	
C5B	−0.3244 (2)	1.1093 (2)	1.64970 (18)	0.0288 (4)	
H5B	−0.3137	1.0430	1.7028	0.035*	
C6B	−0.2177 (2)	1.09262 (19)	1.54106 (17)	0.0244 (4)	
H6B	−0.1335	1.0154	1.5212	0.029*	
C9	0.6997 (2)	0.5511 (2)	0.82747 (19)	0.0296 (4)	
H9A	0.7349	0.6020	0.7657	0.035*	
H9B	0.7592	0.5220	0.8789	0.035*	
C10	0.7045 (2)	0.4366 (2)	0.7671 (2)	0.0341 (5)	
H10A	0.6483	0.4675	0.7130	0.041*	
H10B	0.6607	0.3918	0.8299	0.041*	
C11	0.8511 (3)	0.3413 (3)	0.6941 (3)	0.0277 (5)	0.732 (3)
H11A	0.8469	0.2631	0.6684	0.033*	0.732 (3)
H11B	0.9089	0.3156	0.7470	0.033*	0.732 (3)
C12	0.9238 (3)	0.3919 (3)	0.5817 (2)	0.0290 (4)	0.732 (3)
H12A	0.8692	0.4133	0.5263	0.035*	0.732 (3)
H12B	0.9250	0.4721	0.6063	0.035*	0.732 (3)
C13	1.0739 (3)	0.2945 (3)	0.5141 (3)	0.0291 (4)	0.732 (3)
H13A	1.0747	0.2090	0.5059	0.035*	0.732 (3)
H13B	1.1049	0.3203	0.4316	0.035*	0.732 (3)
C14	1.1788 (4)	0.2821 (3)	0.5752 (3)	0.0300 (4)	0.732 (3)
H14A	1.1396	0.2724	0.6621	0.036*	0.732 (3)
H14B	1.1915	0.3627	0.5697	0.036*	0.732 (3)
C15	1.3220 (3)	0.1684 (3)	0.5202 (3)	0.0312 (4)	0.732 (3)
H15A	1.3815	0.1636	0.5680	0.037*	0.732 (3)
H15B	1.3094	0.0877	0.5267	0.037*	0.732 (3)
C16	1.3971 (4)	0.1766 (4)	0.3891 (3)	0.0338 (5)	0.732 (3)
H16A	1.4981	0.1246	0.3693	0.051*	0.732 (3)
H16B	1.3789	0.2666	0.3757	0.051*	0.732 (3)
H16C	1.3633	0.1440	0.3372	0.051*	0.732 (3)
C11X	0.8421 (8)	0.3668 (9)	0.6579 (8)	0.0285 (6)	0.268 (3)
H11C	0.8566	0.2765	0.6465	0.034*	0.268 (3)
H11D	0.8273	0.4080	0.5845	0.034*	0.268 (3)
C12X	0.9771 (7)	0.3637 (7)	0.6639 (6)	0.0290 (5)	0.268 (3)
H12C	0.9746	0.4507	0.6527	0.035*	0.268 (3)
H12D	0.9824	0.3424	0.7453	0.035*	0.268 (3)
C13X	1.1062 (8)	0.2679 (8)	0.5704 (7)	0.0292 (5)	0.268 (3)
H13C	1.0997	0.2891	0.4893	0.035*	0.268 (3)
H13D	1.1081	0.1812	0.5822	0.035*	0.268 (3)
C14X	1.2442 (9)	0.2622 (8)	0.5726 (7)	0.0304 (5)	0.268 (3)
H14C	1.2471	0.2498	0.6557	0.036*	0.268 (3)
H14D	1.2477	0.3456	0.5516	0.036*	0.268 (3)
C15X	1.3729 (10)	0.1538 (9)	0.4851 (8)	0.0316 (5)	0.268 (3)
H15C	1.3658	0.0711	0.5020	0.038*	0.268 (3)
H15D	1.4577	0.1471	0.4992	0.038*	0.268 (3)
C16X	1.3887 (11)	0.1750 (11)	0.3520 (8)	0.0325 (6)	0.268 (3)
H16D	1.4877	0.1310	0.3017	0.049*	0.268 (3)
H16E	1.3540	0.2676	0.3426	0.049*	0.268 (3)
H16F	1.3345	0.1401	0.3266	0.049*	0.268 (3)
H2N	0.391 (3)	0.9588 (18)	1.044 (2)	0.039*	

### Atomic displacement parameters (Å^2^)

**Table:**

	*U*^11^	*U*^22^	*U*^33^	*U*^12^	*U*^13^	*U*^23^
P1	0.0189 (2)	0.0175 (2)	0.0191 (2)	−0.00565 (18)	−0.00590 (18)	−0.00221 (17)
S1	0.0163 (2)	0.0228 (2)	0.0291 (3)	−0.00693 (18)	−0.00338 (18)	−0.00559 (18)
S2	0.0158 (2)	0.0252 (2)	0.0388 (3)	−0.00881 (19)	−0.0055 (2)	−0.0017 (2)
N1	0.0158 (7)	0.0212 (7)	0.0198 (7)	−0.0079 (6)	−0.0047 (6)	0.0030 (6)
N2	0.0159 (7)	0.0207 (8)	0.0233 (8)	−0.0081 (6)	−0.0032 (6)	−0.0009 (6)
C1	0.0182 (8)	0.0156 (8)	0.0168 (8)	−0.0061 (7)	−0.0061 (7)	0.0019 (6)
C2	0.0170 (8)	0.0236 (9)	0.0210 (9)	−0.0061 (7)	−0.0044 (7)	−0.0019 (7)
C3	0.0205 (9)	0.0280 (10)	0.0236 (9)	−0.0127 (8)	−0.0084 (7)	0.0029 (7)
C4	0.0267 (9)	0.0211 (9)	0.0195 (9)	−0.0114 (8)	−0.0097 (7)	0.0014 (7)
C5	0.0200 (9)	0.0187 (8)	0.0165 (8)	−0.0053 (7)	−0.0043 (7)	0.0001 (7)
C6	0.0171 (8)	0.0152 (8)	0.0156 (8)	−0.0058 (7)	−0.0055 (7)	0.0035 (6)
C7	0.0188 (8)	0.0183 (8)	0.0174 (8)	−0.0070 (7)	−0.0051 (7)	0.0009 (7)
C8	0.0181 (8)	0.0218 (9)	0.0188 (9)	−0.0079 (7)	−0.0056 (7)	0.0017 (7)
C1A	0.0177 (8)	0.0166 (8)	0.0160 (8)	−0.0046 (7)	−0.0018 (7)	−0.0031 (6)
C2A	0.0192 (9)	0.0255 (9)	0.0254 (9)	−0.0089 (8)	−0.0049 (7)	−0.0014 (7)
C3A	0.0280 (10)	0.0274 (10)	0.0306 (11)	−0.0161 (8)	−0.0035 (8)	0.0022 (8)
C4A	0.0275 (10)	0.0222 (9)	0.0232 (10)	−0.0081 (8)	−0.0019 (8)	0.0027 (7)
C5A	0.0223 (9)	0.0249 (9)	0.0214 (9)	−0.0051 (8)	−0.0062 (7)	−0.0011 (7)
C6A	0.0210 (9)	0.0208 (9)	0.0213 (9)	−0.0080 (7)	−0.0047 (7)	−0.0025 (7)
C1B	0.0233 (9)	0.0217 (9)	0.0180 (8)	−0.0093 (7)	−0.0075 (7)	−0.0026 (7)
C2B	0.0295 (10)	0.0206 (9)	0.0223 (9)	−0.0068 (8)	−0.0050 (8)	0.0003 (7)
C3B	0.0292 (11)	0.0275 (10)	0.0268 (10)	−0.0067 (9)	−0.0017 (8)	−0.0050 (8)
C4B	0.0330 (11)	0.0373 (11)	0.0194 (9)	−0.0182 (9)	−0.0023 (8)	−0.0029 (8)
C5B	0.0404 (12)	0.0321 (11)	0.0205 (9)	−0.0194 (9)	−0.0136 (9)	0.0050 (8)
C6B	0.0278 (10)	0.0227 (9)	0.0239 (9)	−0.0092 (8)	−0.0119 (8)	0.0003 (7)
C9	0.0179 (9)	0.0297 (10)	0.0315 (11)	−0.0047 (8)	−0.0017 (8)	−0.0103 (8)
C10	0.0303 (11)	0.0306 (11)	0.0379 (12)	−0.0156 (9)	−0.0027 (9)	−0.0054 (9)
C11	0.0291 (8)	0.0263 (8)	0.0242 (9)	−0.0103 (7)	−0.0065 (7)	0.0001 (7)
C12	0.0290 (8)	0.0285 (7)	0.0249 (8)	−0.0094 (6)	−0.0069 (6)	−0.0004 (6)
C13	0.0285 (8)	0.0301 (7)	0.0248 (8)	−0.0096 (7)	−0.0077 (7)	−0.0009 (6)
C14	0.0287 (8)	0.0320 (7)	0.0256 (7)	−0.0103 (7)	−0.0079 (7)	−0.0006 (6)
C15	0.0282 (8)	0.0351 (7)	0.0265 (9)	−0.0106 (7)	−0.0082 (7)	0.0002 (7)
C16	0.0282 (9)	0.0390 (8)	0.0280 (10)	−0.0099 (7)	−0.0074 (8)	0.0006 (8)
C11X	0.0290 (9)	0.0283 (9)	0.0249 (10)	−0.0101 (8)	−0.0073 (8)	−0.0015 (8)
C12X	0.0288 (8)	0.0294 (7)	0.0250 (8)	−0.0100 (7)	−0.0073 (7)	−0.0008 (7)
C13X	0.0284 (8)	0.0305 (7)	0.0251 (8)	−0.0100 (7)	−0.0077 (7)	−0.0006 (7)
C14X	0.0283 (9)	0.0332 (8)	0.0260 (9)	−0.0104 (8)	−0.0078 (8)	−0.0001 (7)
C15X	0.0284 (9)	0.0352 (8)	0.0265 (9)	−0.0103 (7)	−0.0077 (8)	0.0000 (7)
C16X	0.0285 (9)	0.0370 (9)	0.0268 (10)	−0.0104 (8)	−0.0076 (9)	0.0000 (9)

### Geometric parameters (Å, º)

**Table:**

P1—C1B	1.8322 (19)	C9—C10	1.505 (3)
P1—C1A	1.8325 (18)	C9—H9A	0.9900
P1—C1	1.8481 (18)	C9—H9B	0.9900
S1—C8	1.7391 (19)	C10—C11	1.513 (4)
S1—C9	1.8120 (19)	C10—C11X	1.559 (8)
S2—C8	1.6744 (18)	C10—H10A	0.9900
N1—C7	1.281 (2)	C10—H10B	0.9900
N1—N2	1.378 (2)	C11—C12	1.531 (4)
N2—C8	1.337 (2)	C11—H11A	0.9900
N2—H2N	0.898 (17)	C11—H11B	0.9900
C1—C2	1.398 (3)	C12—C13	1.529 (4)
C1—C6	1.409 (2)	C12—H12A	0.9900
C2—C3	1.386 (3)	C12—H12B	0.9900
C2—H2	0.9500	C13—C14	1.526 (4)
C3—C4	1.388 (3)	C13—H13A	0.9900
C3—H3	0.9500	C13—H13B	0.9900
C4—C5	1.382 (3)	C14—C15	1.529 (4)
C4—H4	0.9500	C14—H14A	0.9900
C5—C6	1.400 (2)	C14—H14B	0.9900
C5—H5	0.9500	C15—C16	1.511 (4)
C6—C7	1.467 (2)	C15—H15A	0.9900
C7—H7	0.9500	C15—H15B	0.9900
C1A—C2A	1.394 (3)	C16—H16A	0.9800
C1A—C6A	1.398 (3)	C16—H16B	0.9800
C2A—C3A	1.391 (3)	C16—H16C	0.9800
C2A—H2A	0.9500	C11X—C12X	1.524 (9)
C3A—C4A	1.382 (3)	C11X—H11C	0.9900
C3A—H3A	0.9500	C11X—H11D	0.9900
C4A—C5A	1.391 (3)	C12X—C13X	1.501 (8)
C4A—H4A	0.9500	C12X—H12C	0.9900
C5A—C6A	1.384 (3)	C12X—H12D	0.9900
C5A—H5A	0.9500	C13X—C14X	1.528 (9)
C6A—H6A	0.9500	C13X—H13C	0.9900
C1B—C6B	1.392 (3)	C13X—H13D	0.9900
C1B—C2B	1.394 (3)	C14X—C15X	1.529 (9)
C2B—C3B	1.392 (3)	C14X—H14C	0.9900
C2B—H2B	0.9500	C14X—H14D	0.9900
C3B—C4B	1.381 (3)	C15X—C16X	1.534 (10)
C3B—H3B	0.9500	C15X—H15C	0.9900
C4B—C5B	1.385 (3)	C15X—H15D	0.9900
C4B—H4B	0.9500	C16X—H16D	0.9800
C5B—C6B	1.385 (3)	C16X—H16E	0.9800
C5B—H5B	0.9500	C16X—H16F	0.9800
C6B—H6B	0.9500		
			
C1B—P1—C1A	104.27 (8)	C11—C10—H10A	108.8
C1B—P1—C1	101.59 (8)	C9—C10—H10B	108.8
C1A—P1—C1	102.39 (8)	C11—C10—H10B	108.8
C8—S1—C9	102.85 (9)	H10A—C10—H10B	107.7
C7—N1—N2	114.84 (15)	C10—C11—C12	114.6 (2)
C8—N2—N1	120.21 (15)	C10—C11—H11A	108.6
C8—N2—H2N	120.0 (17)	C12—C11—H11A	108.6
N1—N2—H2N	119.0 (17)	C10—C11—H11B	108.6
C2—C1—C6	118.18 (16)	C12—C11—H11B	108.6
C2—C1—P1	121.83 (13)	H11A—C11—H11B	107.6
C6—C1—P1	119.94 (13)	C13—C12—C11	112.7 (2)
C3—C2—C1	121.53 (17)	C13—C12—H12A	109.1
C3—C2—H2	119.2	C11—C12—H12A	109.1
C1—C2—H2	119.2	C13—C12—H12B	109.1
C2—C3—C4	119.95 (17)	C11—C12—H12B	109.1
C2—C3—H3	120.0	H12A—C12—H12B	107.8
C4—C3—H3	120.0	C14—C13—C12	114.3 (2)
C5—C4—C3	119.64 (17)	C14—C13—H13A	108.7
C5—C4—H4	120.2	C12—C13—H13A	108.7
C3—C4—H4	120.2	C14—C13—H13B	108.7
C4—C5—C6	120.99 (16)	C12—C13—H13B	108.7
C4—C5—H5	119.5	H13A—C13—H13B	107.6
C6—C5—H5	119.5	C13—C14—C15	113.8 (2)
C5—C6—C1	119.71 (16)	C13—C14—H14A	108.8
C5—C6—C7	118.94 (15)	C15—C14—H14A	108.8
C1—C6—C7	121.30 (16)	C13—C14—H14B	108.8
N1—C7—C6	120.42 (16)	C15—C14—H14B	108.8
N1—C7—H7	119.8	H14A—C14—H14B	107.7
C6—C7—H7	119.8	C16—C15—C14	113.9 (3)
N2—C8—S2	120.86 (14)	C16—C15—H15A	108.8
N2—C8—S1	114.09 (13)	C14—C15—H15A	108.8
S2—C8—S1	125.05 (11)	C16—C15—H15B	108.8
C2A—C1A—C6A	118.71 (17)	C14—C15—H15B	108.8
C2A—C1A—P1	116.43 (14)	H15A—C15—H15B	107.7
C6A—C1A—P1	124.85 (14)	C15—C16—H16A	109.5
C3A—C2A—C1A	120.56 (18)	C15—C16—H16B	109.5
C3A—C2A—H2A	119.7	H16A—C16—H16B	109.5
C1A—C2A—H2A	119.7	C15—C16—H16C	109.5
C4A—C3A—C2A	120.29 (19)	H16A—C16—H16C	109.5
C4A—C3A—H3A	119.9	H16B—C16—H16C	109.5
C2A—C3A—H3A	119.9	C12X—C11X—C10	118.2 (6)
C3A—C4A—C5A	119.53 (18)	C12X—C11X—H11C	107.8
C3A—C4A—H4A	120.2	C10—C11X—H11C	107.8
C5A—C4A—H4A	120.2	C12X—C11X—H11D	107.8
C6A—C5A—C4A	120.40 (18)	C10—C11X—H11D	107.8
C6A—C5A—H5A	119.8	H11C—C11X—H11D	107.1
C4A—C5A—H5A	119.8	C13X—C12X—C11X	113.2 (7)
C5A—C6A—C1A	120.51 (18)	C13X—C12X—H12C	108.9
C5A—C6A—H6A	119.7	C11X—C12X—H12C	108.9
C1A—C6A—H6A	119.7	C13X—C12X—H12D	108.9
C6B—C1B—C2B	118.33 (18)	C11X—C12X—H12D	108.9
C6B—C1B—P1	116.22 (14)	H12C—C12X—H12D	107.7
C2B—C1B—P1	125.33 (15)	C12X—C13X—C14X	115.0 (7)
C3B—C2B—C1B	120.41 (19)	C12X—C13X—H13C	108.5
C3B—C2B—H2B	119.8	C14X—C13X—H13C	108.5
C1B—C2B—H2B	119.8	C12X—C13X—H13D	108.5
C4B—C3B—C2B	120.60 (19)	C14X—C13X—H13D	108.5
C4B—C3B—H3B	119.7	H13C—C13X—H13D	107.5
C2B—C3B—H3B	119.7	C13X—C14X—C15X	113.0 (7)
C3B—C4B—C5B	119.42 (19)	C13X—C14X—H14C	109.0
C3B—C4B—H4B	120.3	C15X—C14X—H14C	109.0
C5B—C4B—H4B	120.3	C13X—C14X—H14D	109.0
C4B—C5B—C6B	120.14 (19)	C15X—C14X—H14D	109.0
C4B—C5B—H5B	119.9	H14C—C14X—H14D	107.8
C6B—C5B—H5B	119.9	C14X—C15X—C16X	112.8 (8)
C5B—C6B—C1B	121.09 (18)	C14X—C15X—H15C	109.0
C5B—C6B—H6B	119.5	C16X—C15X—H15C	109.0
C1B—C6B—H6B	119.5	C14X—C15X—H15D	109.0
C10—C9—S1	107.87 (14)	C16X—C15X—H15D	109.0
C10—C9—H9A	110.1	H15C—C15X—H15D	107.8
S1—C9—H9A	110.1	C15X—C16X—H16D	109.5
C10—C9—H9B	110.1	C15X—C16X—H16E	109.5
S1—C9—H9B	110.1	H16D—C16X—H16E	109.5
H9A—C9—H9B	108.4	C15X—C16X—H16F	109.5
C9—C10—C11	113.6 (2)	H16D—C16X—H16F	109.5
C9—C10—C11X	113.2 (4)	H16E—C16X—H16F	109.5
C9—C10—H10A	108.8		
			
C7—N1—N2—C8	177.59 (16)	C3A—C4A—C5A—C6A	0.0 (3)
C1B—P1—C1—C2	23.69 (17)	C4A—C5A—C6A—C1A	0.4 (3)
C1A—P1—C1—C2	−83.93 (16)	C2A—C1A—C6A—C5A	−0.6 (3)
C1B—P1—C1—C6	−153.73 (14)	P1—C1A—C6A—C5A	−179.15 (14)
C1A—P1—C1—C6	98.65 (14)	C1A—P1—C1B—C6B	−177.27 (14)
C6—C1—C2—C3	−0.6 (3)	C1—P1—C1B—C6B	76.58 (15)
P1—C1—C2—C3	−178.08 (14)	C1A—P1—C1B—C2B	−1.38 (19)
C1—C2—C3—C4	1.0 (3)	C1—P1—C1B—C2B	−107.53 (17)
C2—C3—C4—C5	−0.6 (3)	C6B—C1B—C2B—C3B	−0.4 (3)
C3—C4—C5—C6	−0.2 (3)	P1—C1B—C2B—C3B	−176.25 (16)
C4—C5—C6—C1	0.6 (3)	C1B—C2B—C3B—C4B	−0.5 (3)
C4—C5—C6—C7	−177.04 (16)	C2B—C3B—C4B—C5B	0.5 (3)
C2—C1—C6—C5	−0.2 (2)	C3B—C4B—C5B—C6B	0.3 (3)
P1—C1—C6—C5	177.34 (13)	C4B—C5B—C6B—C1B	−1.3 (3)
C2—C1—C6—C7	177.37 (16)	C2B—C1B—C6B—C5B	1.3 (3)
P1—C1—C6—C7	−5.1 (2)	P1—C1B—C6B—C5B	177.49 (15)
N2—N1—C7—C6	176.72 (15)	C8—S1—C9—C10	−170.42 (15)
C5—C6—C7—N1	−12.7 (3)	S1—C9—C10—C11	−175.95 (19)
C1—C6—C7—N1	169.78 (16)	S1—C9—C10—C11X	163.0 (4)
N1—N2—C8—S2	179.22 (13)	C9—C10—C11—C12	−67.3 (3)
N1—N2—C8—S1	−1.6 (2)	C11X—C10—C11—C12	25.7 (11)
C9—S1—C8—N2	176.61 (14)	C10—C11—C12—C13	177.4 (2)
C9—S1—C8—S2	−4.25 (15)	C11—C12—C13—C14	−75.4 (3)
C1B—P1—C1A—C2A	117.75 (14)	C12—C13—C14—C15	169.6 (3)
C1—P1—C1A—C2A	−136.70 (14)	C13—C14—C15—C16	62.2 (4)
C1B—P1—C1A—C6A	−63.64 (17)	C9—C10—C11X—C12X	33.4 (8)
C1—P1—C1A—C6A	41.91 (17)	C11—C10—C11X—C12X	−62.1 (11)
C6A—C1A—C2A—C3A	0.4 (3)	C10—C11X—C12X—C13X	165.7 (7)
P1—C1A—C2A—C3A	179.05 (15)	C11X—C12X—C13X—C14X	179.6 (7)
C1A—C2A—C3A—C4A	0.0 (3)	C12X—C13X—C14X—C15X	173.9 (7)
C2A—C3A—C4A—C5A	−0.2 (3)	C13X—C14X—C15X—C16X	66.9 (10)

### Hydrogen-bond geometry (Å, º)

**Table:**

*D*—H···*A*	*D*—H	H···*A*	*D*···*A*	*D*—H···*A*
N2—H2*N*···S2^i^	0.90 (2)	2.45 (2)	3.3337 (17)	168 (2)

Symmetry code: (i) −*x*+1, −*y*+2, −*z*+2.
